# Superior Mesenteric Artery Syndrome After Previous Nuss Procedure: A Case Report of Anatomical Realignment With Unintended Consequences

**DOI:** 10.1155/crgm/2887106

**Published:** 2026-06-29

**Authors:** Pin Wen Wang, Chun-Hsiang Wang, Hui-Ting Hsu, Yi-Wei Chou

**Affiliations:** ^1^ School of Medicine, China Medical University, Taichung, Taiwan, cmu.edu.cn; ^2^ Department of Hepatogastroenterology, Tainan Municipal Hospital (Managed by Show Chwan Medical Care Corporation), Tainan, Taiwan, tmh.org.tw; ^3^ Department of Radiology, Tainan Municipal Hospital (Managed by Show Chwan Medical Care Corporation), Tainan, Taiwan, tmh.org.tw; ^4^ Department of Surgery, Tainan Municipal Hospital (Managed by Show Chwan Medical Care Corporation), Tainan, Taiwan, tmh.org.tw

**Keywords:** duodenojejunostomy, Nuss procedure, pectus excavatum, superior mesenteric artery syndrome

## Abstract

**Background:**

Superior mesenteric artery (SMA) syndrome is a rare condition resulting from compression of the third segment of the duodenum. The condition is associated with a reduction of the aortomesenteric angle and distance between the SMA and the abdominal aorta, often due to weight loss or anatomical changes. The Nuss procedure, a minimally invasive corrective procedure for pectus excavatum, may inadvertently alter this anatomy.

**Case Presentation:**

We report a case of a 22‐year‐old male with a history of pectus excavatum who presented with two weeks of abdominal pain and vomiting. Abdominal A CT scan and esophagogastroduodenoscopy examinations were performed, revealing duodenal compression with a narrowed aortomesenteric distance of 6 mm, which is consistent with SMA syndrome. The patient then underwent laparoscopic duodenojejunostomy anastomosis bypass after failed conservative management with complete symptom resolution.

**Conclusion:**

Clinicians should be aware of the potential development of SMA syndrome in patients following the Nuss procedure. Monitoring nutritional status and prompt evaluation of gastrointestinal symptoms are essential to early diagnosis and intervention.

## 1. Introduction

Superior mesenteric artery (SMA) syndrome, also known as Wilkie’s syndrome, is a rare cause of proximal bowel obstruction, resulting from external compression between the abdominal aorta and the SMA occluding the third segment of the duodenum. SMA syndrome has an estimated incidence rate of around 0.013%–0.78% [1]. While people of all ages can be affected, 75% of cases are reported to be in patients aged 10–39 years old. [1, 2]. Due to its rarity and diagnostic challenges, delayed treatments and complications can be associated with significant mortality and morbidity [3].

Loss of retroperitoneal fat predisposes patients to duodenal compression by reducing the aortomesenteric angle to less than 25° and the aortomesenteric distance to less than 10 mm [4, 5]. Such depletion of fatty tissues can be caused by severe weight loss, which can be exacerbated by a variety of conditions such as adolescent growth spurt, anorexia, malabsorptive diseases, malnutrition, hypermetabolism from drugs and burns, and cachexia‐causing conditions including malignancy and tuberculosis. Congenital anatomical abnormalities include excessively low origin of SMA, abnormally high duodenal insertion at the ligament of Treitz, or malrotation of the intestine and SMA. Acquired causes that lead to duodenal compression include lengthening of the spine from scoliosis correctional surgery, intestinal surgery such as ileal pouch‐anal anastomosis (IPAA) or colectomy, body cast, and aging [1, 4, 6]. However, 40.4% of SMA syndrome cases are idiopathic [2].

The Nuss procedure is a standard minimally invasive procedure for pectus excavatum, a congenital chest wall deformity characterized by sternal compression [7]. Around 80% cases of pectus excavatum are male, and around 15%–20% of cases are associated with scoliosis. While pectus excavatum often presents at birth, most patients do not notice the morphological characteristics until the adolescent growth spurt [8]. The severity of the deformity can be measured through the Haller index, in which indices greater than 3.25 are generally categorized as severe and may need surgical repair [9]. While the Nuss procedure is effective for restoring chest wall symmetry, the elevation of the sternum and potential weight loss may inadvertently decrease the aortomesenteric angle, increasing the risk of SMA syndrome. This report discusses a rare case of SMA syndrome following the Nuss procedure and highlights its diagnostic and therapeutic considerations.

## 2. Case Presentation

A 22‐year‐old male patient with a prior history of pectus excavatum repair (via the Nuss procedure 7 years prior) presented to the emergency room with abdominal pain and vomiting that had persisted for two weeks. He also reported intermittent diarrhea but denied fever or recent travel. Despite prior outpatient evaluation and treatment, his symptoms persisted.

### 2.1. Initial Evaluation

On examination, the patient was alert and oriented. The patient’s vital signs upon arrival were as follows: a temperature of 36.7°C, heart rate of 102 bpm, respiratory rate of 18/min, and blood pressure of 119/85 mmHg. Abdominal examination revealed periumbilical tenderness without rebound tenderness; bowel sounds were hyperactive, and the extremities were unremarkable.

Laboratory tests were also conducted on the same day; the results were a creatinine of 1.0 mg/dL, lipase of 19 U/L, potassium of 3.79 mEq/L, glucose of 142 mg/dL, sodium of 136.0 mEq/L, alanine transaminase (ALT) of 18 U/L, aspartate transaminase (AST) of 30 U/L, white blood cell count of 9030/μL (67% segmented neutrophils and 25% lymphocytes), hemoglobin of 16.2 g/dL, and platelet count of 312,000/μL. Most laboratory results were within normal limits except for hyperglycemia and neutrophilia. A chest X‐ray showed no active lung lesions, and a plain abdominal X‐ray revealed no evidence of ileus.

### 2.2. Imaging and Diagnosis

Due to persistent vomiting despite initial medical treatment, a contrast‐enhanced computed tomography (CT) scan of the whole abdomen was performed (Figure 1.). An upper gastrointestinal series (Figure 2) showed a dilated stomach and duodenal bulb and a narrowing at the location of the third portion of the duodenum. Esophagogastroduodenoscopy examination (Figure 3) was also performed, showing an incomplete narrowing of the duodenum lumen at the third portion. The findings suggested gastric distention and the third portion duodenum compression by the SMA with a narrow aortomesenteric distance (6 mm), raising concern for possible gastric outlet obstruction. Nasogastric tube insertion was recommended for gastric decompression. However, the patient was unable to tolerate the procedure. The patient had also tried various other conservative measures such as left lateral decubitus and prone and knee‐to‐chest positions with minimal to no improvements. Based on the clinical findings and imaging results, the patient underwent laparoscopic duodenojejunostomy anastomosis bypass. The patient resumed oral intake on postoperative day 7 and was discharged on Day 13 with complete resolution of gastrointestinal symptoms.

**FIGURE 1:**
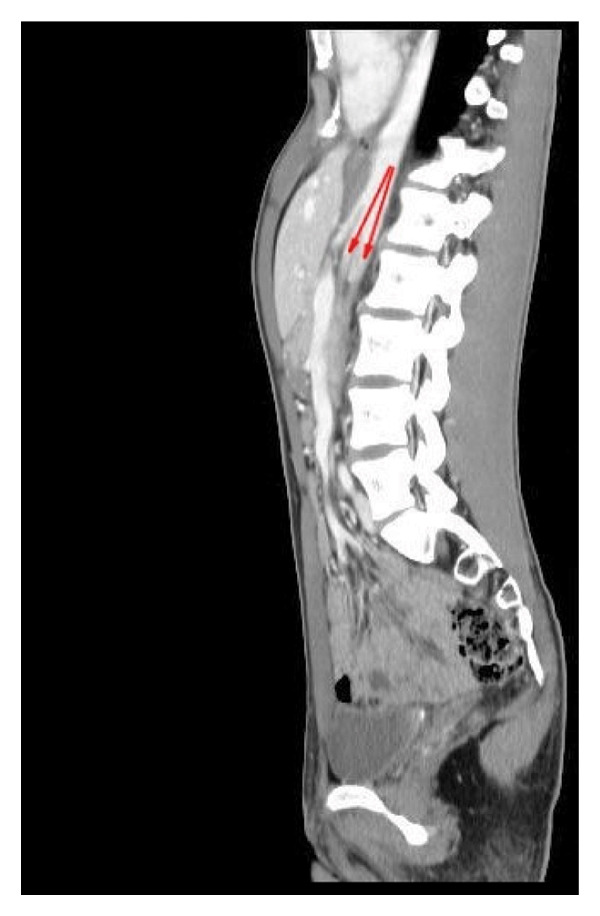
CT angiography with sagittal reconstruction demonstrated an aortomesenteric angle of 10° and aortomesenteric distance of 6 mm. Red arrows indicate the SMA (anterior arrow) and abdominal aorta (posterior arrow). Duodenal compression is visible between the vessels. There was a depletion of fatty tissue surrounding the superior mesenteric artery.

**FIGURE 2:**
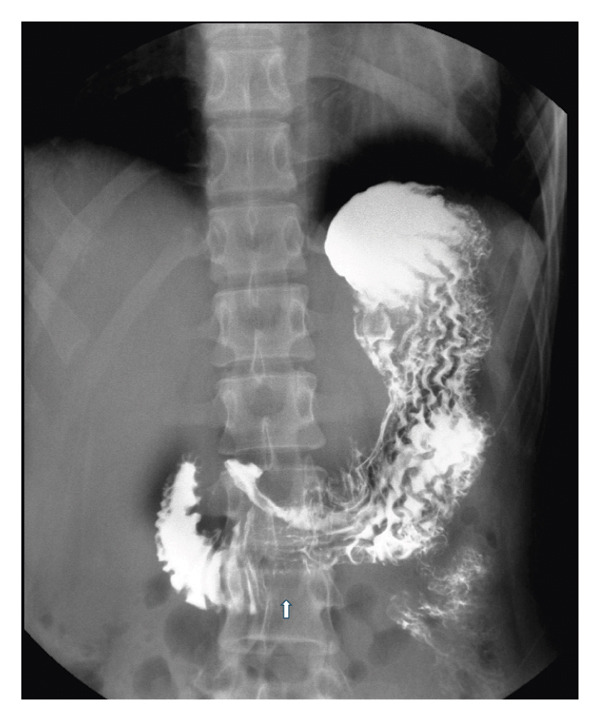
Upper gastrointestinal series reveals a dilated stomach and duodenal bulb, with narrowing at the third portion of the duodenum (arrow).

**FIGURE 3:**
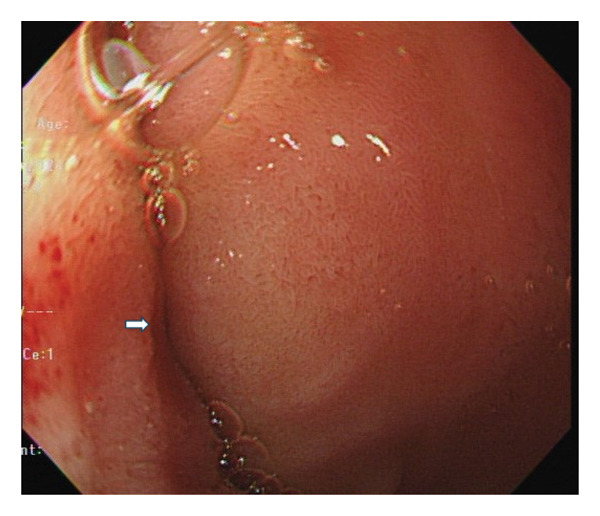
Esophagogastroduodenoscopy examination shows partial luminal narrowing of the third portion of the duodenum (arrow).

## 3. Discussion

SMA syndrome poses a diagnostic challenge due to its nonspecific presentation. Symptoms such as nausea, bilious vomiting, early satiety, weight loss, and epigastric pain can mimic other gastrointestinal conditions [1, 6]. Symptoms alleviated by adopting certain postural positions, such as the knee‐to‐chest, face‐down, or left lateral decubitus position, in which the aortomesenteric angle is released, may provide diagnostic clues [1, 4].

Diagnosis of SMA syndrome requires a combination of both clinical symptoms and imaging findings. X‐ray (both plain film and barium studies), angiogram, CT scans, abdominal ultrasound (US), magnetic resonance imaging (MRI), endoscopy, gastric‐emptying scintigraphy, and multichannel manometry may be used for diagnosis. CT scan remains the gold standard for diagnosis as it allows for accurate measurement of the aortomesenteric angle and distance. X‐ray films may show dilation of the duodenum with constriction near the SMA [1, 4]. Laboratory results are not diagnostic, as electrolyte, protein, and albumin levels may still be within the normal range even with weight loss [1].

Conservative measures are usually recommended for initial treatment, including duodenum and stomach decompression therapy and weight gain. Decompression of the duodenum and stomach can be done through postural change and/or nasogastric tube suction with intravenous metoclopramide as an aid to decompression. Weight gain restores the cushion between the SMA and aorta by increasing retroperitoneal fat. Nasogastric feeding, parenteral feeding, and fluid–electrolyte correction can be considered based on the patient’s needs [1, 4, 10]. A cohort study of 80 SMA syndrome patients between January 2000 and December 2000 by Lee et al. shows that conservative treatments for SMA syndrome have success rates of 71.3% with recurrence rates of 15.8%; 18.7% of the SMA syndrome patients require surgical intervention [11]. While there is no definitive timing for the transition to surgical treatments, a study conducted by Shin and Kim on children with SMA syndrome recommends at least 6 weeks of conservative treatments before considering surgical interventions [12].

Surgical treatments are recommended when patients fail to show clinical or nutritional improvement after a period of conservative management. Indications include continued weight loss, abdominal pain, nausea, vomiting, and dilation of the duodenum. Surgical interventions are recommended especially in patients who are elderly with a history of abdominal operations, immobile, have progressive and/or chronic symptoms, or have arteriosclerosis of SMA [1]. Various surgical options may include duodenojejunostomy, gastrojejunostomy, gastroduodenostomy, Strong’s procedure, Ladd’s procedure, and anterior transposition of the duodenum’s third segment, among others. A recent large cohort study of 91 SMA syndrome patients who underwent laparoscopic duodenojejunostomy between February 2016 and July 2023 by Yetişir et al. indicates that the surgical procedure yields high success rates (93.4% of patients with symptoms improvements) with minimal postoperative complications [13]. Laparoscopic duodenojejunostomy has since replaced open bypass surgery as the preferred surgical option, reinforcing the role of the minimally invasive bypass surgery as the standard treatment of SMA syndrome [1, 4].

The Nuss procedure involves the retrosternal placement of one or more curved metal bars to the sternum anteriorly to correct pectus excavatum, increasing the anteroposterior thoracic diameter [14, 15]. Several mechanisms may explain the occurrence of SMA syndrome after the Nuss procedure. It can be hypothesized that the surgical correction stretches the anterior abdominal wall and pulls the mesenteric root superiorly and posteriorly. The alteration in thoracic and potentially abdominal architecture may inadvertently change the tension on the mesentery or the position of the abdominal aorta relative to the spine, thereby unmasking SMA syndrome by pushing the sternum forward, resulting in narrowing the aortomesenteric angle and decreasing the aortomesenteric distance. The procedure also induces postural realignment after correction of severe pectus excavatum, which may change the relative orientation of the SMA and abdominal aorta [16]. Lastly, postoperative weight loss may also lead to the loss of retroperitoneal fat, which may further exacerbate the reduction of the aortomesenteric angle. These changes may predispose patients to SMA syndrome [17]. Although rare, clinicians should maintain a high index of suspicion in patients presenting with upper GI symptoms post‐Nuss repair, particularly if weight loss is present.

## 4. Conclusion

This case underscores the importance of recognizing SMA syndrome as a potential complication following the Nuss procedure. Anatomical adjustment from the Nuss procedure combined with weight loss may predispose patients to reduced aortomesenteric angle and distance, leading to duodenal compression. Early recognition and prompt intervention are crucial to prevent complications and achieve favorable outcomes. Nutritional monitoring, patient postoperative education, and long‐term monitoring after chest wall surgery are recommended to ensure early intervention.

## Author Contributions

Pin Wen Wang, Chun‐Hsiang Wang, and Yi‐Wei Chou contributed equally to this study.

## Funding

This work did not receive financial support.

## Conflicts of Interest

The authors declare no conflicts of interest.

## Data Availability

All relevant clinical information is within the article; no additional datasets were generated. Patient confidentiality restricts sharing of raw data.
